# Acute chest syndrome in sickle cell disease/HBE patient, A case report

**DOI:** 10.1002/ccr3.4575

**Published:** 2021-08-21

**Authors:** Ibrahim Khamees, Waail Rozi, Mohamed A. Yassin

**Affiliations:** ^1^ Department of Internal medicine Hamad Medical Corporation Doha Qatar; ^2^ Department of Medical Oncology/Hematology Section Hamad Medical Corporation Doha Qatar

**Keywords:** acute chest syndrome, HBSE, hemoglobin E, hemoglobin S, sickle cell disease

## Abstract

The presented case will shed some light on one of the rarest complications of HBSE disease, which is acute chest syndrome, and will highlight the management of that complication.

## Graphical abstract

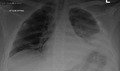

## 1 INTRODUCTION

Hemoglobin S (HBS) and hemoglobin E (HBE) are common hemoglobinopathies, but combined heterozygosity of HBS and HBE (HBSE) is relatively a rare disease. Most reports suggest that HBSE is generally a benign condition with few reported complications like vaso‐occlusive crises, acute chest syndrome, and bone necrosis. In this report, we describe a 17‐year‐old male patient presenting with acute chest syndrome treated with exchange transfusion.

HBS, which is the hemoglobin type responsible for sickle cell disease, is localized mostly in Africa, India, and some parts of the Middle East.^1^ In sickle cell disease, there is homozygosity of HBS mutation, and in stressful conditions like infections hemoglobin will be deoxygenated and HBS will polymerize in the vessels, leading to multiple complications including vaso‐occlusive pain and acute chest syndrome.^2^ HBE is found mostly in Southeast Asia. Patients who are homozygous for HBE generally have mild anemia and are clinically asymptomatic, whereas individuals who are heterozygotes for HBE and Beta‐thalassemia, for example, have a variable clinical spectrum ranging from mild to very severe disease requiring regular blood transfusion.^3^ Due to the increasing rate of migrations and marriages happening between different races, increasing numbers of patients with compound heterozygosity for HBS and HBE are found worldwide, and scarce data about the disease and the complications related to it have been reported.

Acute chest syndrome is considered the most common cause of death in sickle cell disease patients. It is defined by the presence of new radiographic pulmonary infiltrates as well as fever and respiratory symptoms. Management ranges from pain control and fluid replacement in mild disease to simple transfusion or exchange transfusion in more severe presentations.^4^ Acute chest syndrome mostly occurs in patients who are homozygous for HBS, however, there are few reported cases of acute chest syndrome happening in patients with HBSE disease.^5^, ^6^

## 2 CASE REPORT

A 17‐year‐old Arabic male patient who is known to have HBSE disease, presented with a history of chest and back pain for few days prior to admission. He also complained of a non‐productive cough for 2 days but denied fever. On examination, his heart rate was 110 and his respiratory rate was around 25 with a normal temperature of 36.9. On chest exam, he had left basal crackles with decreased air entry.

Initial laboratory investigations are mentioned in Table 1.

**TABLE 1:** laboratory investigations

Investigation	Result	Normal range
WBC	9.5 ×10^3/μl	(4–10) ×10^3/μl
RBC	5.5 ×10^6/μl	(3.8–4.8) ×10^6/μl
Hb	13.3 gm/dl	(12–15) gm/dl
HCT	38.1%	(36–46) %
MCV	69.4 fl	(83–101) fl
PLT	205 ×10^3/μl	(150–400) ×10^3/μl
CRP	152.8 mg/L	(0–5) mg/L
Lactic acid	1.3 mmol/L	(0.5–2.2) mmol/L
Procalcitonin	0.08 ng/ml	<2 ng/ml

**FIGURE 1:**
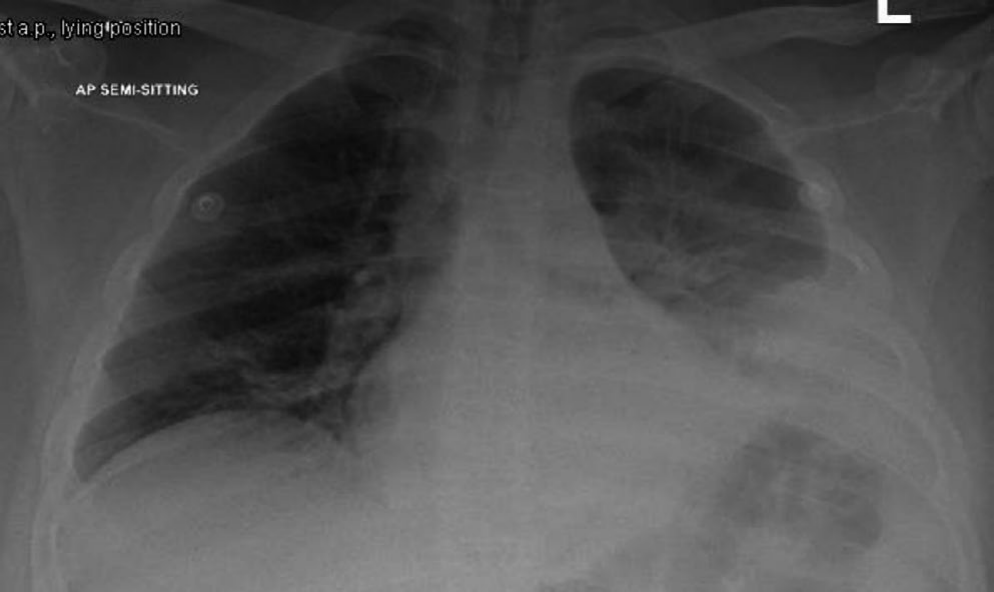
chest x‐ray

His chest x‐ray showed left retrocardiac and left lower lung zone opacity suggesting left lower lobe consolidation, shown in (Figure 1).

The patient was diagnosed to have community‐acquired pneumonia and started on ceftriaxone 1000 mg daily for seven days and azithromycin 500 mg daily for three days as an inpatient. The patient did not improve and started requiring oxygen two days after admission, he became more tachypneic and tachycardiac so he was shifted to the medical intensive care unit and started on non‐invasive ventilation, COVID‐19 and all cultures were negative. Hematology recommended doing hemoglobin electrophoresis for the patient considering the possibility of acute chest syndrome. Hemoglobin electrophoresis was done and it showed the findings in Table 2.

**TABLE 2:** Hemoglobin electrophoresis

Hemoglobin type	Percentage
Hb A	00
Hb A2	2.0
Hb F	2.2
Hb S	67.1
Hb E	28.7
Sickle test	Positive

The patient underwent a session of exchange transfusion, then during the following days, the patient improved gradually and was discharged from the hospital 4 days after the exchange transfusion. He was commenced on folic acid 5 mg daily and hydroxyurea 500 mg twice a day as prophylaxis, with close follow‐up in hematology clinics.

## 3 DISCUSSION

HBS molecule is a result of the replacement of the hydrophilic glutamic acid by the hydrophobic valine residue at position 6 in the beta‐globin chain. This mutation results in the sickle shape of the affected hemoglobin molecule, which changes to that shape when deoxygenated in certain situations like infections.^7^ This process can cause several acute complications like acute vaso‐occlusive pain, acute chest syndrome, stroke, and priapism.^8^, ^9^ Sickle cell disease can result in some chronic complications as well, for example, it can cause chronic anemia, chronic bone pain, and avascular necrosis.^10^

HBE molecule was first identified and reported independently in 1954.^11^ Its synthesis is caused by a base substitution at codon 26 of the beta‐globin gene, GAG‐AAG, which ends up in the substitution of lysine for glutamic acid. It can be co‐inherited with several hemoglobinopathies such as beta‐thalassemia. The compound heterozygosity for HbE and beta‐thalassemia, HbE beta‐thalassemia, is a remarkably heterogeneous disease with a diverse clinical spectrum ranging from mild anemia to the most severe forms of beta‐thalassemia major.^3^

HBSE disease on the other hand is considered a benign condition in general. However, David Masiello et al.^12^, in their review described 26 patients with HBSE heterozygosity, 9 of them had clear complications attributed to sickling ranging from acute painful episodes to splenic thrombosis and acute chest syndrome, all of these patients were above 20‐year‐old. They suggested also that patients with HBSE disease should be followed and managed in the same way as patients with Hb S/b1^+^ thalassemia.

Sickling associated complications in HBSE disease are thought to occur more commonly in people aged more than 20, but some reports described young patients with this disease who had severe complications as well. A review was done in turkey (An area with a high rate of consanguinity) reported 20 patients with HBSE disease, two of them had mild vaso‐occlusive crises and one who was diagnosed with the disease at age of 11 and was started on hydroxyurea, suffered from cerebrovascular stroke later on.^13^ Another case report described a young girl who was diagnosed with HBSE disease at the age of 5 due to leg pain, where the images suggested bone infarction. At the age of 7, she had a fatal massive bone marrow embolism.^14^

One of the organs that is commonly affected in sickle cell disease patients is the eye. Many complications can occur including anterior and posterior segments ischemia, iris infarction, Erythrocyte‐induced glaucoma which may occur in sickle cell disease patients following traumatic or post‐surgical hyphema, and proliferative sickle retinopathy, which in particular happens more in milder genotypes like SC or S‐B thalassemia than in the SS genotype.^15^ Interestingly, one patient with HBSE disease was reported to have proliferative sickle retinopathy and treated with sector laser photocoagulation.^16^ Another patient with HBSE disease presented with traumatic hyphema who then developed repeated bleeds in the anterior chamber, elevated intraocular pressure, and bilateral reversible sickle cell retinopathy.^17^

Acute chest syndrome is uncommon to happen in patients with HBSE disease. Upon reviewing the literature, we found 5 cases of HBSE disease complicated by acute chest syndrome.^5^, ^6^, ^18^, ^19^, ^20^ In one case report, a 66‐year‐old patient with HBSE disease suffered from bone infarction and acute chest syndrome which improved with simple blood transfusion of two units along with supportive care and was discharged on hydroxyurea.^5^ Another case report presented a 22‐year‐old patient with HBSE disease diagnosed with parvovirus B19 infection and acute chest syndrome, also received simple blood transfusion and improved.^6^ A third paper described a 24‐year‐old patient with HBSE disease admitted with acute chest syndrome and managed with supportive therapy only.^20^ Our patient was admitted with acute chest syndrome which did not resolve with antibiotics and required exchange transfusion, after which the patient improved significantly and was discharged on hydroxyurea.

## 4 CONCLUSION

HBSE disease is thought to be a benign condition, but more and more reports are being published describe complicated HBSE disease. An increased number of cases of HBSE disease is expected with the increasing rate of migrations and interracial marriages. This warrants close follow‐up of such patients and early recognition and management of the complications of this disease especially the severe ones like acute chest syndrome.

## CONFLICTS OF INTEREST

The authors report no conflicts of interest in this work.

## AUTHOR CONTRIBUTIONS

Ibrahim Khamees: manuscript writing and literature review. Waail rozi: case presentation writing. Mohamed A Yassin: mentorship, manuscript writing, and literature review.

## ETHICAL APPROVAL

Written informed consent was obtained from the patient to allow the publication of information including images. Case approved by HMC Medical Research Center.

## DATA AVAILABILITY STATEMENT

Data available on request due to privacy/ethical restrictions.
